# Loneliness associates with endothelial dysfunction in a community-based cohort: a pilot study and translational approach

**DOI:** 10.1038/s44325-025-00059-5

**Published:** 2025-06-26

**Authors:** Yvonne Baumer, Brian A. Tirado, Lola R. Ortiz-Whittingham, Andrew S. Baez, Cristhian A. Gutierrez-Huerta, Laurel G. Mendelsohn, Abhinav Saurabh, Joshua A. Jacobs, Marcus R. Andrews, Valerie M. Mitchell, Billy S. Collins, Antwan Jones, Daniella M. Schwartz, Anca D. Dobrian, Michael V. Stanton, Tiffany M. Powell-Wiley

**Affiliations:** 1 Social Determinants of Obesity and Cardiovascular Risk Laboratory, Cardiovascular Branch, Division of Intramural Research, National Heart, Lung and Blood Institute, Bethesda, MD USA; 2 Department of Sociology and Department of Epidemiology, The George Washington University, Washington, DC USA; 3 Division of Rheumatology and Clinical Immunology, University of Pittsburgh, Pittsburgh, PA USA; 4 Department of Biomedical and Translational Sciences, Eastern Virginia Medical School at Old Dominion University, Norfolk, VA USA; 5 Department of Public Health, California State University, East Bay, Hayward, CA USA; 6 Intramural Research Program, National Institute on Minority Health and Health Disparities, NIH, Bethesda, MD USA

**Keywords:** Cardiovascular biology, Cardiovascular biology

## Abstract

Loneliness is known to be an important contributor to cardiovascular disease (CVD). However, little is known about the impact of loneliness on endothelial barrier integrity, a crucial hallmark of CVD development and progression. In this study, we aimed to investigate how loneliness might impact the endothelium. We found greater perceived loneliness associated with the product of circulating epinephrine and TNFα (E/T), which, in turn, associated with lower VE-cadherin on endothelial cells in an ex vivo experiment. Additionally, circulating plasma levels of soluble VE-cadherin were associated significantly with subclinical CVD. To explore the mechanistic aspects of these associations, we measured the effects of E/T on endothelial barrier function in vitro. E/T treatment decreased endothelial VE-cadherin expression and dampened endothelial barrier integrity, involving at least partial JAK/Stat signaling, highlighting a potential additive effect of epinephrine and TNFα on endothelial dysfunction, potentially accelerating CVD development and progression in individuals experiencing loneliness-related chronic stress.

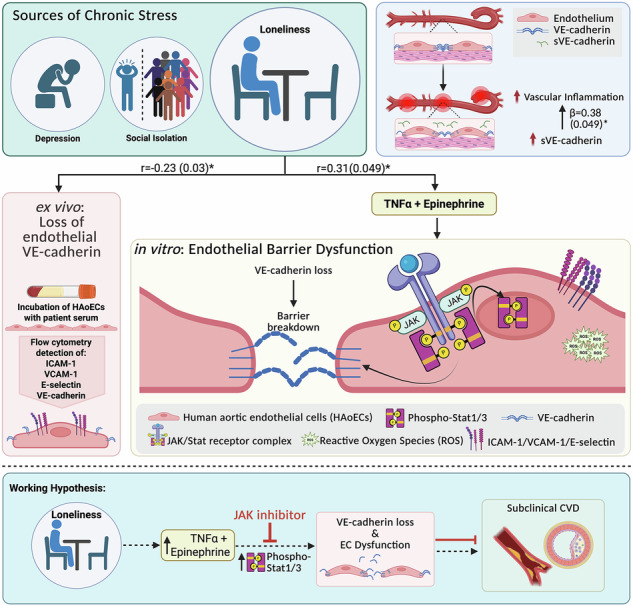

## Introduction

Cardiovascular disease (CVD) remains among the leading cause of death in the United States (US)^1^, impacting racial and ethnic minorities disproportionally^2^, and worsened by chronic psychosocial and environmental stressors^3,4^. Loneliness and social isolation are significant chronic psychosocial stressors. In March 2023, the US Office of the Surgeon General published an advisory on the importance of addressing loneliness and social isolation as a public health threat^5^. Loneliness refers to the subjective feeling of being alone despite having social connections. In contrast, social isolation describes the lack of social connection^6^. More than 30% of adults over 45 report being lonely, and 25% of adults 65 and older are considered socially isolated^7^. Both loneliness and social isolation have been associated with increased risk of hypertension and obesity^8^, both risk factors for CVD. A recent study with more than 55,000 US adults enrolled in the Women’s Health Initiative Extension Study II reported an increased risk for incident CVD in lonely or socially isolated individuals, with the strongest association observed in women experiencing loneliness and social isolation together^9^. Undoubtedly, loneliness and social isolation are deeply connected and intertwined with depression in a likely bi-directional fashion^10–12^ and are also associated with the increasing presence of CV risk factors and incident CVD^13^. Taken together, these findings underscore the need to further understand the mechanisms through which loneliness and social isolation contribute to CVD.

The specific signaling mechanisms by which chronic psychosocial stressors, either alone or together, elevate CVD risk are currently under thorough investigation^14^. Chronic stress activates the hypothalamic-pituitary-adrenal (HPA) axis and sympathetic nervous system (SNS), increasing cortisol and catecholamines (norepinephrine and epinephrine), as noted by Rohleder^15^. This response is associated with elevated levels of pro-inflammatory cytokines.

Endothelial dysfunction, an early marker of atherogenesis^16^, can be detected before structural changes in the vasculature occur^17^ and is thought to be impacted by chronic stress^18^. Endothelial dysfunction is a multifaceted pathology with characteristics that include alterations in nitric oxide (NO) homeostasis^19^; increased expression of intercellular adhesion molecules like ICAM-1, VCAM-1, or E-selectin^20^ which are known to facilitate transmigration of immune cells into the subendothelial space; and loss of the endothelial barrier integrity^21^. While evidence indicates that processes involving NO production^22^ and intercellular adhesion molecule expression^23^ are altered by chronic stress, little is known about the impact of chronic stress on endothelial barrier integrity. Vascular endothelial-cadherin (VE-cadherin) is an essential inter-endothelial adherens junction molecule that has a key role in maintaining vascular integrity^24^. Recent studies by Okoh et al. (2023) and O’Sullivan et al. (2023) highlight the impact of stress on endothelial function in vivo, measured by flow-mediated dilation (FMD). These studies identified transient endothelial dysfunction (TED) and found TED to be particularly severe in Black individuals, increasing CVD risk by 41.9%. Nearly 70% of this increased risk is linked to compromised endothelial function under stress^25^. Additionally, these studies suggest that catecholamines significantly contribute to the stress-related decline in FMD^26^.

An important gap remains in our understanding of the long-term vascular impacts of elevated catecholamine and cytokine levels, alone or together, on the endothelium and, in particular, on endothelial barrier integrity in humans. Furthermore, the combined effect of these molecules and endothelial barrier dysfunction under chronic stress is not well understood. Therefore, we aimed to evaluate a potential biological pathway through which chronic psychosocial stress might drive CVD risk associated with endothelial cell (EC) dysfunction in a community-based cohort of AA individuals. First, we investigated the relationship between chronic psychosocial stressors and cytokine or catecholamine levels in a potentially synergistic manner as well as markers of EC dysfunction, using an ex vivo approach. Second, we performed in vitro experiments to evaluate the impact of cytokine/catecholamine synergism on human EC barrier function and explored the underlying signaling mechanisms involved. Lastly, we hypothesized that soluble serum VE-cadherin, indicative of endothelial injury, is associated with subclinical CVD measured by vascular inflammation (VI) from ^18^FDG-PET/CT.

## Results

### Cohort of African American participants from resource-limited neighborhoods

The cohort included 42 AAs from under-resourced neighborhoods of the Washington, D.C. area with at least one CVD risk factor (Supplementary Table 1). The study participants have a median of 60.6 years of age, are mainly female, and, on average, had class I obesity (average BMI of 33). The study participants had an average loneliness score of 32.45 (range: 23–52), social isolation score of 0.40 (range: 0–2), and depression score of 3.71 (range: 0–25). Loneliness (*r* = 0.43, *p* = 0.007) and social isolation correlated significantly with depression (*r* = 0.53, *p* = 0.001).

### Synergism of epinephrine and TNFα associated with greater self-reported loneliness in African American participants from resource-limited neighborhoods

Here, we explored the product of each cytokine with each catecholamine as a measure of synergism (Table 1 and Supplementary Table 2). Our analysis focused on IL-1β and TNFα, particularly, as both of these cytokines, in previous studies in this patient cohort by our laboratory, have been shown to be associated with neighborhood socioeconomic deprivation and trimethylamine N-oxide (TMAO), as well as epigenetic modulation of stress- and loneliness-related genes^27–29^. We found a positive association between self-reported loneliness levels and the combination of epinephrine and TNFα (*p* = 0.049), while the associations with the individual biomarkers were not statistically significant (Table 1). Nevertheless, all associations were lost after adjustment for multiple comparisons using the Holm-Šídák method.

**Table 1:** Pearson correlation analysis of self-reported measures of psychosocial stressors to IL-1β and TNFα, epinephrine, and each of their products

				Synergism	
	Epi	IL-1β	TNFα	Epi and IL-1β	Epi and TNFα
Social Isolation^a^	−0.21 (0.18) [0.53]	0.21 (0.52) [0.43]	0.10 (0.54) [1.00]	0.17 (0.82) [0.62]	0.01 (1.00) [0.99]
Loneliness^a^	0.08 (0.60) [1.00]	0.04 (1.00) [0.99]	0.25 (0.12) [0.35]	0.05 (1.00) [0.99]	0.31 (0.049)*[0.14]

Results are shown as the Pearson correlation coefficient (*r*) value followed by the p-value in parentheses with the multiple comparison-adjusted *p*-values in brackets. Significance is indicated in bold font and an asterisk (*) for a *p*-value < 0.05 was reached.

^a^Loneliness, Social Isolation – higher values indicates increased chronic psychosocial stress.

### Measures of psychosocial stress associated with markers of endothelial dysfunction in an ex vivo approach

Endothelial dysfunction is a hallmark of CVD, which has been linked in the past to chronic stress. Using an ex vivo approach, we treated human aortic endothelial cells (HAoECs) with cell culture medium supplemented with 10% individual serum samples from each of the 42 study participants (Supplementary Table 1). Cells were treated overnight and processed for flow cytometry to measure the surface expression of ICAM-1, VCAM-1, E-selectin, and VE-cadherin. Expression of these proteins was used as a surrogate marker for endothelial dysfunction and was used in subsequent correlation analyses (Pearson correlation).

Interestingly, only VE-cadherin expression, and not the more traditionally explored markers ICAM-1, VCAM-1, and E-selectin (Table 2), was found to be significantly correlated with loneliness and trended to significance with social isolation. An increase in self-reported social isolation or loneliness was found associated with a loss of VE-cadherin surface expression on HAoECs. This suggests that components within the study participants’ serum altered by chronic psychosocial stressors might be crucial in facilitating this loss of VE-cadherin. However, all associations were lost after multi-comparison adjustment using the Holm-Šídák method.

**Table 2:** Pearson correlation analysis of self-reported measures of psychosocial stressors to HAoEC surface expression of ICAM-1, VCAM-1, E-selectin, and VE-cadherin after overnight incubation with study participants’ serum

	ICAM-1 (MFI)	VCAM-1 (MFI)	E-selectin (MFI)	VE-cadherin (MFI)
**Social Isolation**^a^	−0.05 (0.77) [0.99]	−0.09 (0.56) [0.91]	−0.14 (0.37) [0.75]	−0.28 (0.08) [0.22]
**Loneliness**^a^	−0.21 (0.18) [0.45]	−0.16 (0.33) [0.67]	−0.14 (0.39) [0.77]	−0.23 (0.03)*[0.09]

Results are shown as the Pearson correlation coefficient (*r*) value followed by the *p*-value in parentheses with the multiple comparison-adjusted *p*-values in brackets. Significance is indicated in bold font and an asterisk (*) when a *p*-value < 0.05 was reached.

^a^Loneliness, Social Isolation—higher values indicate increased chronic psychosocial stress; MFI—mean fluorescence intensity units derived from flow cytometry analysis.

### Soluble VE-cadherin associates with subclinical CVD in African Americans

**Fig. 1:**
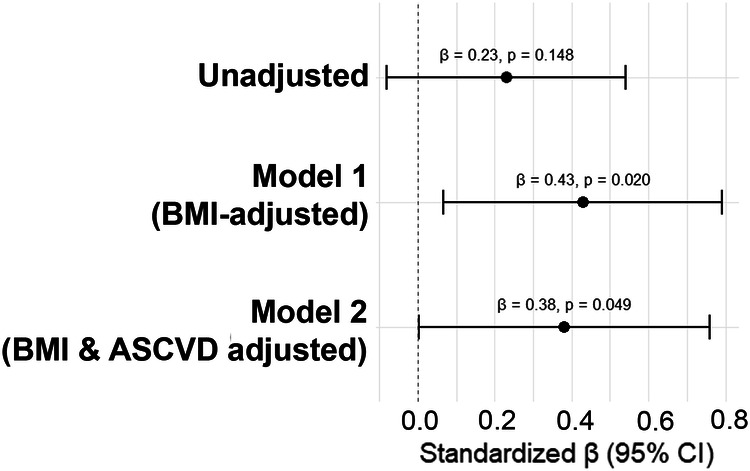
Association between soluble VE-cadherin and vascular inflammation in the DC-CNHA cohort, 2014–2017. Graphical display of the association between soluble VE-cadherin levels as detected by ELISA and Vascular Inflammation as detected by ^18^FDG-PET/CT. Data are shown as Forrest Blot with the standardized beta given, followed by the p-value in parentheses. Models are shown as unadjusted, BMI-adjusted, or BMI plus ASCVD 10-year risk score adjusted values. Significance is indicated by the asterisk and was assumed with a *p*-value reaching <0.05.

VE-cadherin can be shed into the circulation and be detected as soluble VE-cadherin (sVE-cadherin) in serum samples under pathological conditions consistent with endothelial dysfunction. Therefore, we aimed to determine if sVE-cadherin might be associated with subclinical CVD determined as vascular inflammation (VI) measured with ^18^FDG-PET/CT in the cohort of 42 AAs from under-resourced neighborhoods in Washington, D.C. (Supplementary Table 1). In multivariable regression analyses, we found a positive association between VI and sVE-cadherin (*β* = 0.38, *p* = 0.049) after adjustment for BMI and 10-year ASCVD risk score (Table 3, Fig. 1).

**Table 3:** Multivariable linear regression analysis between plasma soluble VE-cadherin levels as detected by ELISA and Vascular Inflammation (VI) as measured by ^18^FDG-PET/CT was performed

	Unadjusted	Model 1	Model 2
Soluble VE-cadherin to Vascular Inflammation	0.23 (0.148)	0.43 (0.020)*	0.38 (0.049)*

Results are shown as the standardized beta (*β*) value followed by the *p*-value in parentheses in the unadjusted, BMI-adjusted model (Model 1), and BMI plus 10-year ASCVD risk score adjusted model (Model 2). Significance is indicated by the asterisk (*).

### Epinephrine exacerbates TNFα-induced loss of endothelial barrier integrity

Our findings indicated that epinephrine (Epi) and TNFα are synergistically associated with self-reported loneliness, which in turn correlated with reduced VE-cadherin expression on HAoECs. However, these associations disappeared after adjusting for multiple comparisons. Consequently, we conducted further experiments to explore whether long-term Epi administration could exacerbate TNFα‘s effects on HAoECs in vitro. We focused on three key indicators of endothelial dysfunction in atherogenesis: increased reactive oxygen species (ROS) formation^20^, upregulation of intercellular adhesion molecules^20^, and the rearrangement of inter-endothelial junction proteins, resulting in compromised endothelial barrier integrity^21^.

First, we explored the potential impact of epinephrine, TNFα, and the combination of epinephrine and TNFα on ROS production (Supplementary Fig. 1a, b). While we did not see an increase in mitochondrial ROS with the combined vs. individual treatments, we found nicotinamide adenine dinucleotide phosphate (NADPH)-dependent ROS formation to be increased with the combination treatment epinephrine + TNFα (Epi+TNFα) (3.11 ± 0.40-fold change of control) when compared to the individual treatments (Epi: 1.93 ± 0.30 and TNFα: 2.06 ± 0.29-fold change of control; Supplementary Fig. 1a, b). Second, we investigated the impact of combination treatment on intercellular adhesion molecules, including ICAM-1, VCAM-1, and E-selectin (Supplementary Fig. 1c–e). The data showed statistically significant enhanced expression of ICAM-1 and E-selectin when treated with the combination of Epi+TNFα, with no changes observed for VCAM-1.

**Fig. 2:**
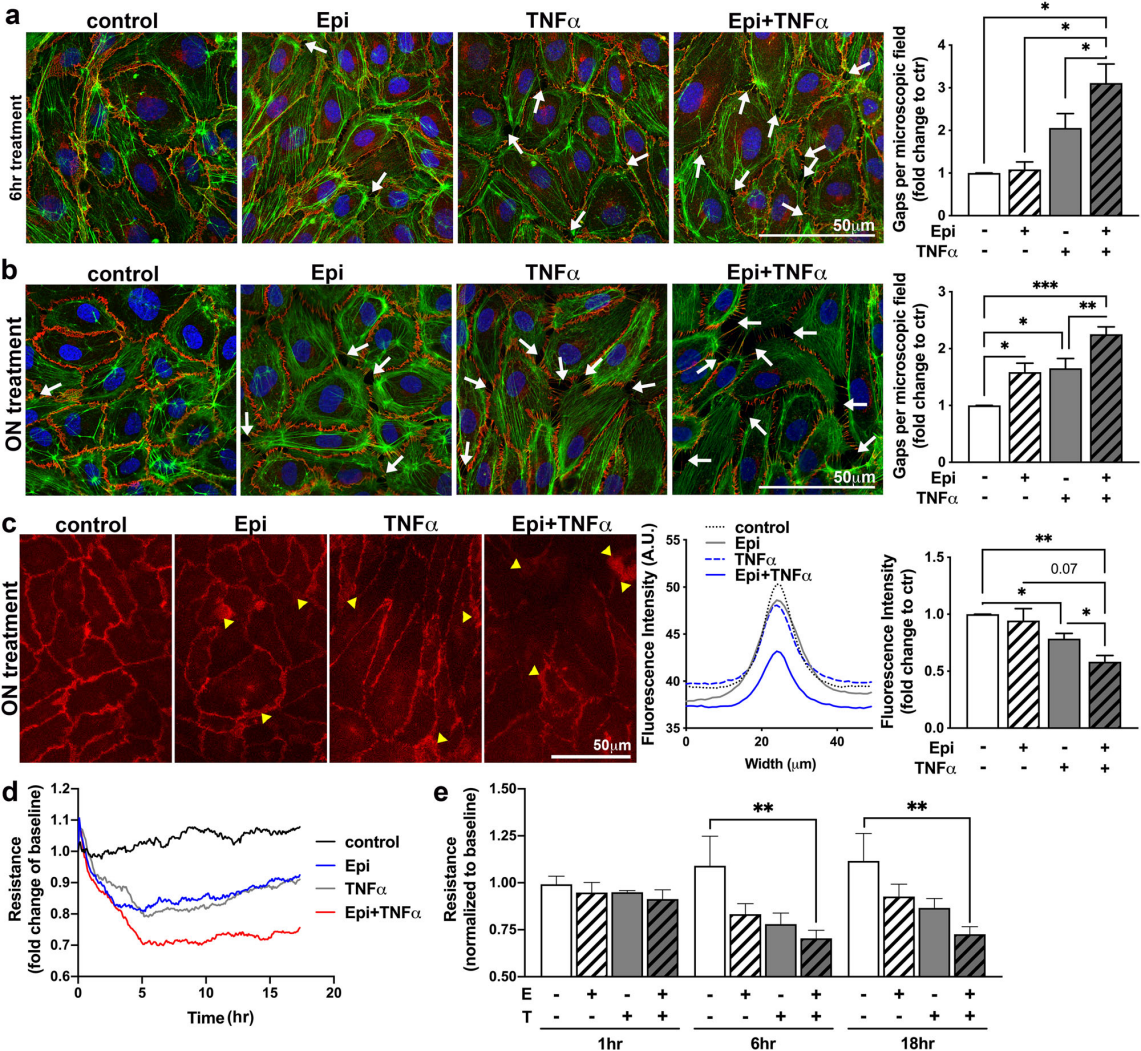
Epinephrine and TNFα treatment combined exacerbate single-treatment effects on endothelial dysfunction. **a**, **b** Immunofluorescence staining of VE-cadherin (red) and labeling of F-actin (green) as well as nuclei (blue) with subsequent quantification of intercellular presence of gaps (6 h: *n* = 5, ON: *n* = 8; RM one-way ANOVA analysis with Tukey correction). Representative images are shown, and gaps are indicated by white arrows. The quantification of intercellular gaps is an indication of endothelial barrier dysfunction, subsequently allowing enhanced transport of blood components and cells into the arterial wall. **c** Images of VE-cadherin immunofluorescence (red) were taken at equal exposure time per experimental set, and the fluorescence intensity over the junctional space was measured as displayed in the histogram. Histogram quantification of VE-cadherin within the inter-endothelial junction allows for quantification of VE-cadherin re-distribution or loss; processes involved in endothelial barrier breakdown. Subsequent quantification of *n* = 5 is displayed as a graph (RM one-way ANOVA analysis with Tukey correction). Additionally, areas of widening and more diffuse VE-cadherin presence are indicated with yellow arrowheads. **d**, **e** HAoECs were subjected to ECIS technology to quantify endothelial barrier integrity. A time curve of the ECIS experiment with indicated treatments and subsequent quantification at indicated time points is shown (*n* = 6; 1 h = RM one-way ANOVA analysis with Tukey correction; 6 h/18 h: Friedman test with Dunn’s correction). (*indicates significance *p* < 0.05, **indicates significance *p* < 0.01; Abbreviations: ECIS electric cell-substrate impedance sensing, Epi epinephrine, HAoEC human aortic endothelial cells, hr hours, ON overnight, RM repeated measures, TNFα tumor necrosis factor-alpha).

Third, we examined the impact of Epi+TNFα treatment on intercellular gaps, VE-cadherin distribution, probably the most essential adhesion junction molecule^24^, and endothelial barrier integrity, as well as recovery after injury (Fig. 2, Supplementary Fig. 1f, g). Treatment of HAoEC monolayers with Epi+TNFα for 6 h resulted in accelerated intercellular presence of gaps when compared to treatment with TNFα alone (Fig. 2a, TNFα: 2.06 ± 0.33 vs. Epi+TNFα: 3.11 ± 0.44-fold change of control) and trending to significance to epinephrine single treatment (p = 0.07). After overnight treatment with epinephrine and TNFα, the cells displayed a significant increase in the presence of gaps (white arrows in Fig. 1a/b), which was again exacerbated when the two treatments were combined (Fig. 2b, Epi: 1.59 ± 0.16 and TNFα: 1.65 ± 0.17 vs Epi+TNFα: 2.25 ± 0.13-fold change of control). Interestingly, we also found changes in VE-cadherin distribution, especially after overnight treatment (Fig. 2c). Further analysis of immunofluorescence images taken at equal exposure times revealed a decreased expression of VE-cadherin within the junctional space when combining Epi + TNFα treatments, as seen by a decrease in fluorescence intensity within the junction (histogram; Epi: 0.94 ± 0.11 and TNFα: 0.79 ± 0.05 vs Epi+TNFα: 0.58 ± 0.06-fold change of control). Additionally, all treatments displayed areas where VE-cadherin staining showed widening, indicating a reorganization of the adherens junctions (yellow arrowheads in Fig. 2c). Subsequently, by utilizing ECIS technology, we were able to observe changes in endothelial barrier integrity over time (Fig. 2d, e), with findings indicating an exacerbated loss in barrier integrity when Epi+TNFα were applied. Interestingly, when HAoEC monolayers were injured after overnight treatment, impaired recovery was observed in the Epi+TNFα-treated monolayers when compared to the single treatment (Supplementary Fig. 1f, g). These data indicate that overnight treatment with Epi+TNFα augments the effects of injury and delays barrier recovery.

### Epinephrine exacerbates TNFα-induced prolonged Stat1/3 activation

**Fig. 3:**
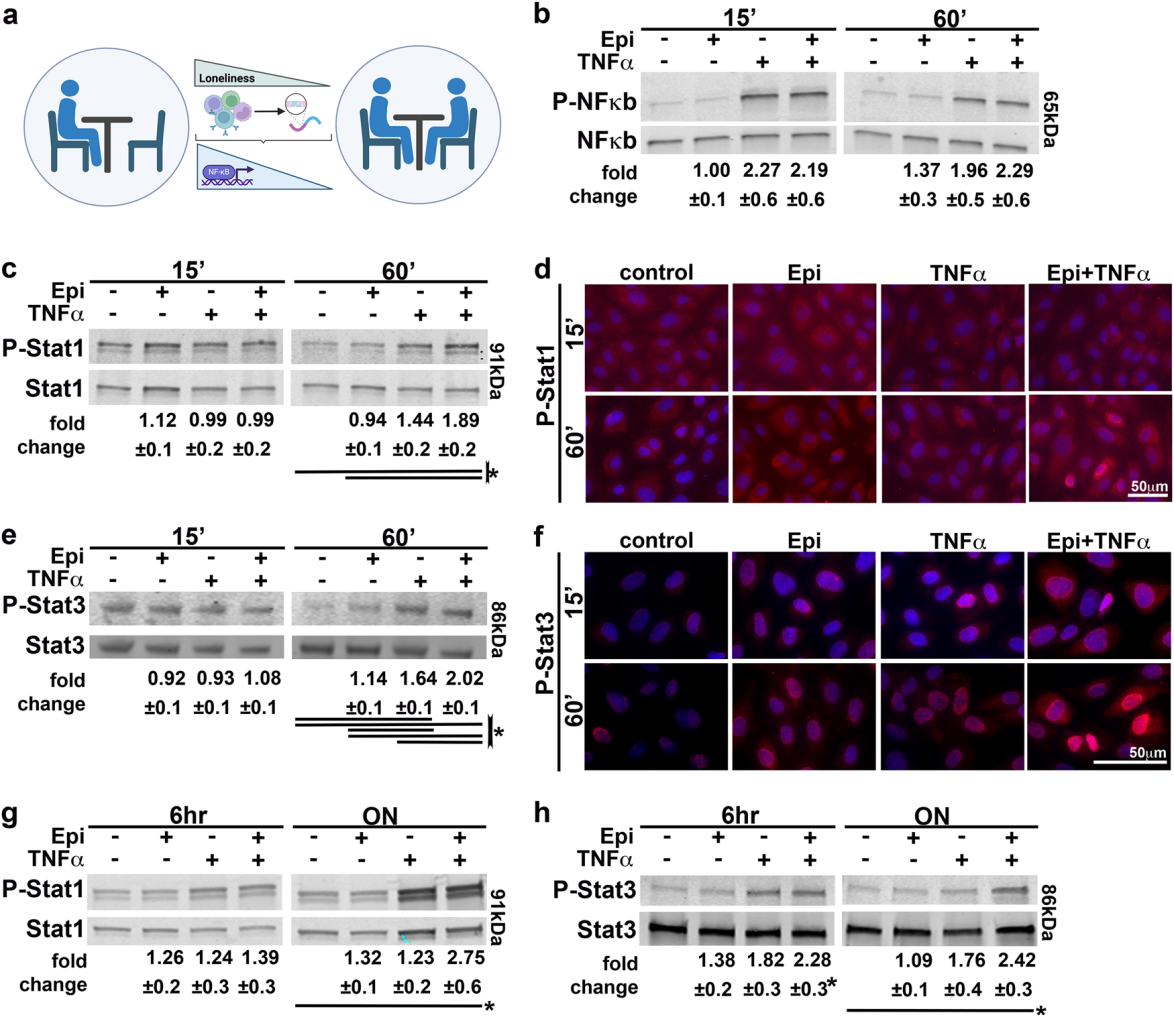
Determination of potential synergistic activation of signaling pathways in Epi + TNF-treated HAoECs. **a** The NFκB pathway has previously been involved in chronic stress- and loneliness-related cell dysfunction, which is graphically summarized. **b–h** HAoECs were treated as shown for the indicated timepoints and subjected to western blot or immunofluorescence analysis. For each analyzed protein, a representative western blot image is shown with the molecular weight indicated on the right of the representative images in kDa. Western blot analysis results are displayed underneath the image. (*n* = 4–5; RM one-way ANOVA analysis with Tukey correction). **b** Western blot results for NFκB activation. **c** Display of Stat1 activation results for 15 and 60 min treatment timepoints. **d** Immunofluorescence staining of HAoECs for the phosphorylated Stat1 (red). Nuclei were labeled using DAPI (blue). (*n* = 3) **e** Presentation of Stat3 activation results for 15 and 60 min treatment timepoints. **f** Immunofluorescence staining of HAoECs for the phosphorylated Stat3 (red) with nuclei being labeled using DAPI in blue. (*n* = 3). **g**, **h** Western blot analysis of long-term activation of Stat1 or Stat3. Representative images are displayed with quantitative analysis results below the image. (Stat1 *n* = 7; Stat3 *n* = 5; RM one-way ANOVA analysis with Tukey correction). (Significance was assumed when *p* < 0.05 and is indicated with the asterisk *; Abbreviations: ‘ minutes, Epi epinephrine, HAoEC human aortic endothelial cells, hr hours, kDA kilodalton, ON overnight, RM repeated measures, TNFα tumor necrosis factor-alpha).

In the next set of experiments, we aimed to determine which signaling pathways are potentially involved in the observed impact of epinephrine on TNFα signaling. Several studies, including our own^27^, have linked chronic stress, particularly loneliness, to activation and alterations of NFκB-related signaling^15,30^, a pathway of key importance for TNFα signaling and atherogenesis^31^ (Fig. 3a). We therefore explored using western blotting whether the potential intracellular-mediating pathway could involve NFκB activation. We found that NFκB activation was not synergistically impacted by the combined treatment of Epi+TNFα (Fig. 3b). However, when determining the activation levels of Stat1 and Stat3, a pathway also highlighted by Cole et al. in leukocytes of individuals with loneliness^30^, we detected an activation of both Stat1 and Stat3 in a delayed manner beyond the individual treatment controls (Fig. 3c–h). While no activation could be observed at 15 min of treatment, a significantly boosted phosphorylation of both proteins could be seen at 60 min of treatment (Fig. 3c, e). Additionally, we were able to localize the phosphorylated states of Stat1 and Stat3 in the nucleus using immunofluorescence, confirming the western blot data at the 60 min treatment timepoint (Fig. 3d, f). Next, we were interested in determining if the activation of Stat1 and Stat3 might be transient or sustained (Fig. 3g, h). Phosphorylation of each of the Stat proteins was detected at increased activation levels after both 6 h and overnight treatments in the combined treatment experiments compared to the individual (especially TNFα), indicating that epinephrine likely exacerbates TNFα-induced activation of Stat1 and Stat3.

### Epinephrine exacerbates TNFα-induced endothelial dysfunction via JAK/Stat signaling

**Fig. 4:**
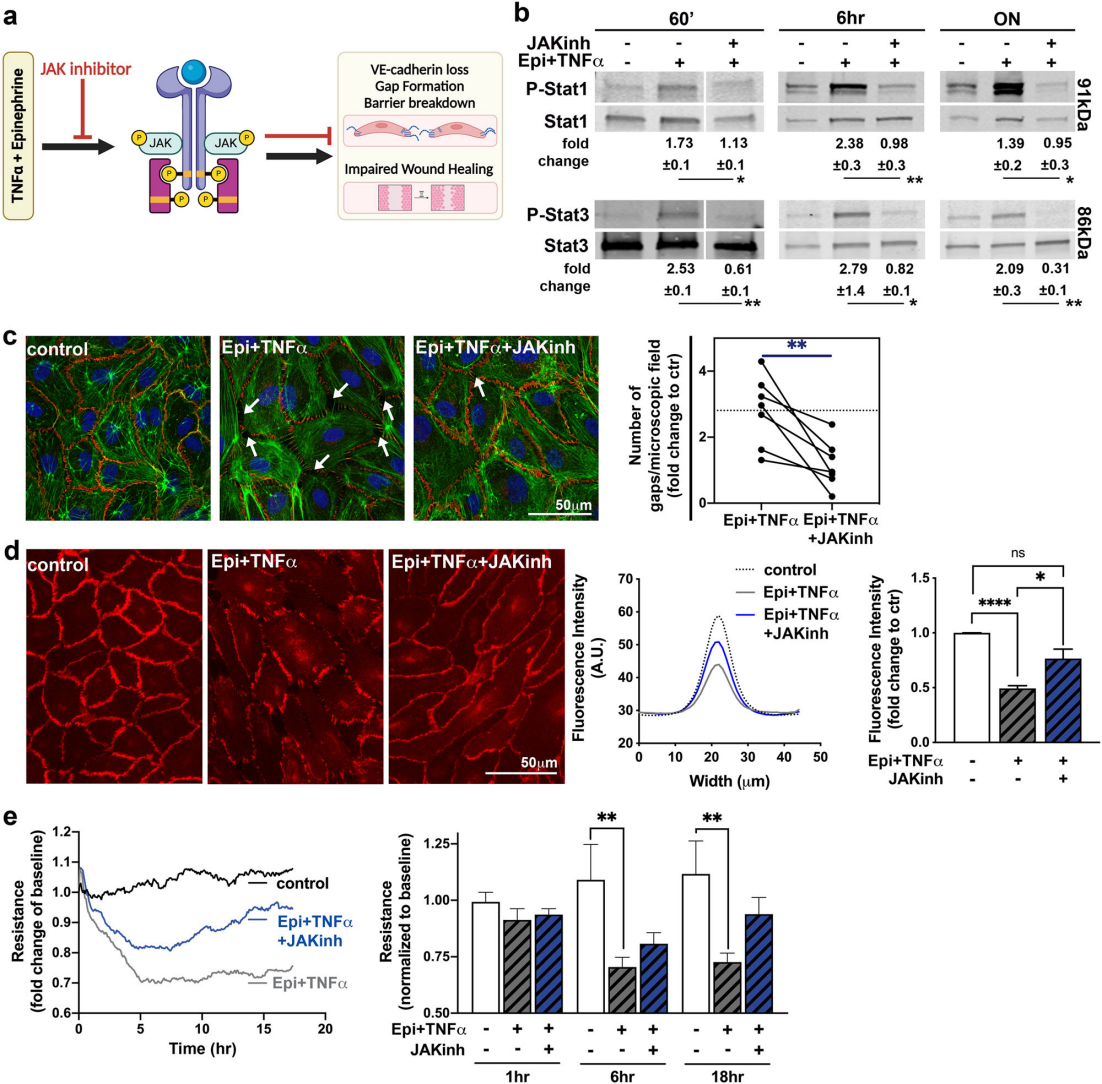
Impact of JAK/Stat pathway inhibition on Epi + TNFα-induced endothelial dysfunction. HAoECs were treated with control, Epi+TNFα, or Epi+TNFα in the presence of tofacitinib, a JAK/Stat pathway inhibitor ON. **a** Summary of the results in this figure and the impact of JAK/Stat inhibition. (**b**) Display of Stat1 and Stat3 western blot results. A representative image of each analyzed protein is shown for each treatment time point. Western blot analysis results are displayed below. (60’: *n* = 4–6; Mixed-effects analysis with Sidak correction; 6 h and ON: *n* = 5; Stat1=RM one-way ANOVA analysis with Tukey correction; Stat3 = Friedman test with Dunn’s correction). **c** Immunofluorescence staining of VE-cadherin (red) and labeling of F-actin (green) as well as the nuclei (blue) with subsequent quantification of intercellular gap formation (*n* = 7; paired t-test between Epi+TNFα and Epi+TNFα + JAK inhibitor). White arrows indicated gaps between endothelial cells. **d** Images of VE-cadherin immunofluorescence after ON treatment were taken at equal exposure time per set, and the fluorescence intensity over the junctional space was measured as displayed in the histogram. Subsequent quantification of *n* = 5 is displayed in the graph (Kruskal-Wallis test with Dunn’s correction). **e** Time curve of ECIS experiment with indicated treatments and subsequent quantification at indicated time points (*n* = 6; Friedman test with Dunn’s correction). (* indicates significance *p* < 0.05, ** indicates significance *p* < 0.01; Abbreviations: ‘minutes, ctr control, ECIS electric cell-substrate impedance sensing, Epi epinephrine, HAoEC human aortic endothelial cells, hr hours, kDA kilodalton, ON overnight, RM repeated measures, TNFα tumor necrosis factor-alpha).

Next, we aimed to determine if the observed long-term activation of Stat1 and Stat3 is dependent on JAK/Stat signaling. We employed tofacitinib, a clinically used JAK inhibitor that has been shown to reduce vascular damage in rheumatoid arthritis patients^32^ (Fig. 4a). First, we determined if the JAK inhibitor Tofacitinib (Tofa) would prevent Epi+TNFα-induced phosphorylation of Stat1/3 (Fig. 4b). Tofa reduced activation of Stat1 and Stat3 significantly below Epi+TNFα levels at 60 min, and the long-term 6 h and overnight treatments.

In a final set of in vitro experiments, we sought to determine if inhibition of JAK/Stat signaling may prevent the impact of Epi+TNFα treatment on endothelial barrier integrity (Fig. 4c–e, Supplementary Fig. 2). First, HAoECs were co-treated for 6 h (Supplementary Fig. 2) and overnight (Fig. 4c–e) with Epi+TNFα and the JAK inhibitor Tofa. We found that inhibition of JAK signaling in the Epi+TNFα treated sample resulted in a 2.48-fold reduction of observed gaps when compared to Epi+TNFα treatment alone (overnight: Epi+TNFα 2.89 ± 0.56 vs JAKinh+ Epi+TNFα 1.17 ± 0.27-fold change to control, p = 0.02). Additionally, when analyzing VE-cadherin staining in the inter-endothelial junction space by immunofluorescence (Fig. 4d), overnight co-treatment with the JAK inhibitor partially rescued the expression of VE-cadherin in the inter-endothelial junctional space. Lastly, when measuring changes in endothelial barrier integrity utilizing ECIS, we found that the addition of the JAK inhibitor also partially rescued the impact of Epi+TNFα on endothelial barrier integrity. These data suggest that JAK/Stat signaling is at least partially responsible for the Epi+TNFα-driven pathological effects on endothelial barrier integrity.

## Discussion

Loneliness has significantly increased over the past decade, especially during the COVID-19 pandemic^5^. Loneliness has been reported to exacerbate CVD risk factors and CVD itself, but the underlying biological mechanisms are largely unknown, especially in a population at disproportionate risk for CVD risk factors and subsequent CV-related deaths. Hence, our study focused on African American individuals residing in under-resourced Washington, D.C. neighborhoods. First, we found that self-reported loneliness, a psychosocial marker of stress, was associated with a loss in HAoEC VE-cadherin expression as well as with a seemingly synergistic relationship between epinephrine and TNFα. We also found that serum sVE-cadherin levels were associated with subclinical CVD in the community-based cohort. Subsequent in vitro studies examining the potential synergism between epinephrine and TNFα revealed that epinephrine enhances TNFα-induced loss of endothelial VE-cadherin and loss of barrier integrity in a JAK/Stat1/3-dependent manner. Our findings suggest that VE-cadherin, an adherens junction protein crucial for endothelial cell function and CVD, may be important in the context of loneliness-related stress among populations disproportionately impacted by adverse social conditions.

sVE-cadherin is associated with a breakdown in endothelial barrier integrity under pro-inflammatory conditions^33^ and has been suggested to be a potential marker of endothelial dysfunction^34^. Here, we demonstrate chronic stress related to loneliness (simulated via the use of overnight treatments) may also drive the shedding of VE-cadherin into the serum. Previous work has supported the idea that sVE-cadherin may be an important biomarker of chronic inflammatory disease activity^35^. Similarly, our findings suggest that sVE-cadherin may be an indicator of VI and subclinical CVD and provide further evidence for the utility of sVE-cadherin as a biomarker of endothelial barrier dysfunction.

Endothelial barrier integrity has also been investigated in the context of the blood-brain barrier (BBB), with VE-cadherin playing an important role in maintaining the BBB^36^. We observed a relationship between chronic stressors and a loss of surface VE-cadherin in HAoECs treated with participants’ sera. Similarly, following chronic stress, VE-cadherin has been demonstrated to be downregulated in mouse nucleus accumbens endothelial cells^36^. Additionally, this and other findings also suggest that stress-induced breakdown in endothelial tight junctions may be related to depression-like behaviors^36,37^. In our cohort, loneliness and depression levels are significantly correlated, emphasizing their close relationship. The link between depression and CVD risk in this community-based cohort, previously suggested by observational data from the study population^38^, is further supported by our data, underscoring the need to consider both depression and endothelial barrier dysfunction in assessing CVD risk.

While previous connections between TNFα and VE-cadherin loss exist^34,36^, the role of synergy between epinephrine and TNFα in the loss of VE-cadherin is less studied. Here, we observed epinephrine exacerbated TNFα-induced loss of VE-cadherin in HAoECs. While epinephrine is thought to benefit endothelial cells under activating conditions^39^, evidence suggests chronic exposure to catecholamines may drive a loss of endothelial barrier integrity in the BBB^36^. In murine microvascular cerebral endothelial cells, treatment with catecholamines was associated with an impaired gene expression of VE-cadherin^40^. Furthermore, the combinatory effect of catecholamines and inflammatory mediators (TNFα and IL-6) is dependent on time, with destruction of the cell monolayer observed after 24 h of treatment^40^. Although this effect was previously only seen in cerebral endothelial cells, these findings, in conjunction with our study, support the need to further investigate the cardiovascular impacts of VE-cadherin loss mediated by biomarkers of stress and inflammatory molecules.

Additionally, epinephrine likely has various effects on endothelial cells, underscoring the need for clinical measures assessing the many aspects of endothelial function. For example, a recent study developed a quantitative magnetic resonance imaging (MRI) workflow to measure arterial endothelial permeability^41^. Developing non-invasive, feasible clinical measures that assess different aspects of endothelial dysfunction will be necessary to determine CVD risk and develop appropriate treatments. Ultimately, the long-term impacts of stress-related elevated epinephrine on endothelial barrier integrity may be deleterious. However, these patterns—along with the potential synergism between epinephrine and TNFα—warrant further investigation.

A recent paper conducted in HUVECs showed that epinephrine induced a dose-dependent reduction in the glycocalyx thickness on ECs, accompanied by EC function loss and increased glycolysis, especially after long-term treatment^42^, two mechanisms also observed in TNFα-treated ECs^43,44^. Interestingly, enhanced glycolysis has been associated with increased presence of ROS^45^ in some pathologies, like cancer, which in turn, in a separate study, has been shown to be capable of Stat1/3 activation^46^. Our results indicate increased ROS presence after long-term exposure to Epi and TNFα, which could potentially contribute to the observed Stat1/3 activation, especially at later timepoints. A separate study investigated the impact of epinephrine on HAECs gene expression^47^. The genes upregulated included genes like FABP4, which has been shown to activate Stat1 in endothelial cells^48^ but has also been shown to regulate intracellular ROS presence^49^. In a recent study, FABP4 protein, as determined by proteomics analyses, was upregulated in individuals with high loneliness and was a partial mediator in the relationship between loneliness and CVD among UK Biobank study participants^50^. Ultimately, it is likely that a variety of independent pathways act synergistically when endothelial cells are exposed to Epi and TNFα simultaneously for prolonged time periods, potentially involving the FABP4–ROS axis and subsequent Stat1/3 activation leading to impairment of endothelial function.

Stat3-mediated endothelial permeability has been shown to be important in sustained loss of endothelial barrier function over time^51^. We observed JAK/Stat signaling was at least partially responsible for the stress-related epinephrine and TNFα-induced loss of VE-cadherin, suggesting additional potential cellular mediators and pathways that may drive VE-cadherin shedding. Research has supported the role of both NFκB and JAK/Stat in stress-related signaling pathways^15^, with previous work revealing an upregulation of the majority of genes in the JAK/Stat signaling pathway in peripheral blood mononuclear cells in response to acute stress and individuals experiencing loneliness^30,52^.

Furthermore, given the role JAK/Stat signaling plays in chronic stress-related loss of VE-cadherin, clinical therapies such as JAK inhibitors may be investigated in the context of endothelial barrier dysfunction and subclinical CVD to determine their potential treatment efficacy. For example, tofacitinib, a JAK inhibitor, attenuated vascular inflammation as measured by ^18^FDG-PET/CT in RA patients at 1-year follow-up^53^, further suggesting JAK/Stat signaling may play a role in vascular inflammation and highlighting the potential of JAK inhibitors to reduce endothelial barrier dysfunction. However, pharmaceutical interventions can result in undesired side effects. In the case of JAK inhibitors, the Food and Drug Administration (FDA) issued a statement requiring a warning to patients for increased risk of cardiovascular events, blood clots, cancers, and even death for some patients^54^. Patients with existing CVD risk factors, like smoking, hypertension, diabetes, age above 65, or with existing CVD or cardiac abnormalities, like arrhythmia, should undergo a risk assessment to evaluate the benefit-to-risk ratio for each patient, severely limiting the patient groups potentially benefiting from these therapeutics^55–57^.

However, our findings emphasize the importance of considering potential behavioral interventions that could mitigate the relationship between chronic stress, sVE-cadherin, and CVD risk. Multi-level stress reduction interventions will be critical in reducing the effects of chronic stress on CVD, particularly among racial and ethnic minoritized populations who may disproportionately experience chronic stressors. Meditation, for example, has been suggested to help reduce CV risk^58^ and is associated with changes in stress-related amygdalar activity and functional connectivity^59,60^. On the neighborhood level, interventions and policies focusing on improving access to place-based resources, e.g. mental health care services^61^, enacting fair housing laws, and equitable neighborhood investments may help minimize stressors to which populations in resource-limited neighborhoods may be exposed^62^. The role of physical activity in reducing CVD risk is also well-known^63^, and previous work supports reduced endothelial dysfunction markers sICAM1 and sE-selectin, in an exercise group compared to a sedentary group^64^. However, the relationship between stress reduction interventions, VE-cadherin, and endothelial barrier integrity needs to be fully understood.

We acknowledge the limitations of our study cohort, which, due to its cross-sectional design, does not allow for the establishment of directionality or causality of the observed associations due to the risk for reverse causation. However, our in vitro data could be seen as proof-of-principle experiments, allowing for careful consideration of our working hypothesis as visualized in our graphical abstract. Additionally, the small sample size does not allow for mediation or moderation analysis, nor examination of the cumulative effects of chronic stressors. Furthermore, the small sample size did not allow for the inclusion of other and potentially more granular adjustments in the statistical models that could confound observed relationships with sVE-cadherin (e.g. diabetes, other chronic conditions). Lastly, while focusing on one particular ethnic or racial group, who undoubtedly is at increased and disproportionate risk for chronic stress and has traditionally been less included in translational or clinical research, could be seen as a limitation, as the generalizability of our data may be argued. Therefore, larger longitudinal studies, including racially and ethnically diverse populations, are needed to confirm our findings, and all our findings should be considered a hypothesis-generating pilot study substantiated by in vitro experiments.

In conclusion, chronic stress is believed to contribute to CVD among racial and ethnic minoritized populations^4^, and here we demonstrate potential biological mechanisms through which chronic stress affects endothelial function to potentially promote poor CVD outcomes among urban African Americans. Taken together, it is crucial to better understand how chronic stress drives adverse CVD outcomes, particularly among populations disproportionately exposed to adverse social conditions, so that targeted interventions can be explored and implemented on the path to health equity.

## Material and methods

### Study participants

The Washington, D.C. Cardiovascular Health and Needs Assessment (DC-CHNA) was an observational, community-based participatory research study. This study aimed to evaluate psychosocial factors and cardiovascular health markers in an AA population at risk for CVD in Washington, D.C., to develop community-based interventions to improve cardiovascular health^65,66^. The DC-CHNA received approval from the National Heart, Lung, and Blood Institute (NHLBI) Institutional Review Board (IRB) (NCT01927783). The NHLBI Institutional Review Board (NCT01143454) approved the secondary study for which 42 of the DC-CHNA study participants were evaluated at the National Institutes of Health (NIH) Clinical Center for additional cardiometabolic phenotyping after providing written informed consent. Complete medical history, physical exam, and surveys for psychosocial measures were completed, and clinical labs were obtained. Additionally, all participants donated blood samples and underwent whole-body ^18^FDG-PET/CT imaging to measure the presence of subclinical CVD as aortic inflammation. Guidelines for good clinical practice were followed according to the Belmont Report (National Commission for the Protection of Human Subjects of Biomedical and Behavioral Research), the Declaration of Helsinki, and the NIH Radiation Safety Commission.

### Stress measurements

Loneliness was measured by the Revised University of California, Los Angeles (UCLA) Loneliness Scale^67^. Participants responded to 20 questions regarding feelings and perceptions of loneliness and companionship. Items were reverse coded as necessary, and response values were summed (range 20–80), with higher scores indicating greater perceived loneliness. Social Isolation was measured utilizing a subscale of the Chronic Stress Scale, a validated questionnaire designed to be divided into 13 subscales^38,68,69^ ranging from 0 to 2, with a higher score indicating higher social isolation.

### Vascular inflammation measurements from ^18^FDG-PET/CT imaging

Whole body ^18^Fluorodeoxyglucose Positron Emission Tomography Computed Tomographic (^18^FDG-PET/CT) images were acquired 1 h after administration of 10 mCi ^18^FDG following an overnight fast of at least 8 h^65,70^. A Siemens Biograph mCT PET/CT 64-slice scanner (Siemens Medical Solutions USA, Malvern, PA, USA) was used to scan cranially to caudally with standard bed positions of 3 min each from the vertex of the skull to the toes^65^.

Aortic vascular inflammation (VI), a marker of subclinical CVD, was measured as previously described^65,71^ by placing regions of interest (ROIs) on 1.5 mm thick axial slices from the aortic root to the bifurcation into the iliac arteries; 10 consecutive ROIs were also placed on the superior vena cava as a measure of background activity. The maximum standardized uptake value (SUV) for each aortic slice was extracted and divided by the mean SUV from the ten background venous measurements. The individual target-to-background ratio (TBR) values for each aortic slice were then averaged to obtain the final TBR measure of aortic VI, our parameter of interest. Increasing VI levels indicate the increasing presence of subclinical CVD.

### Covariates

Body mass index (BMI) was calculated using height and weight measurements taken by our clinical team at the NIH Clinical Center. The atherosclerotic CVD (ASCVD) risk score estimates an individual’s 10-year risk of experiencing an ASCVD event and was calculated using age, race, sex, total cholesterol, high-density lipoprotein cholesterol, blood pressure, and medical history, including diabetes and smoking status^72^.

### Biomarker analysis

Serum and plasma were obtained from our participants. The cytokines, IL-1β and TNFα were measured from EDTA plasma samples as described previously^38,65^. Our analysis focused on IL-1β and TNFα, particularly, as both of these cytokines, in previous studies in this patient cohort by our laboratory, have been shown to be associated with neighborhood socioeconomic deprivation and trimethylamine N-oxide (TMAO), as well as epigenetic modulation of stress- and loneliness-related genes^27–29^. Catecholamines (i.e., epinephrine and norepinephrine) were analyzed from frozen EDTA plasma samples using high-performance liquid chromatography (HPLC) at the Radioimmunoassay and Biomarkers Core at the Institute for Diabetes, Obesity & Metabolism at the University of Pennsylvania (Dr. M. Rickels and Dr. H. W. Collins). sVE-cadherin was measured using the human VE-cadherin Quantikine ELISA kit at the recommended 1:100 dilution per the manufacturer’s recommendation (Cat: DCADV0, R&D Systems, USA).

### Cell culture and stimulation

Primary human aortic endothelial cells (HAoECs, PromoCell, Germany) were cultured using the corresponding media and split kit (PromoCell, Germany) as described previously^73^. Cells were passaged up to 8 times and used when reaching confluence. HAoECs were treated with epinephrine (10 μM, Cayman Chemicals, USA), TNFα (10 ng/ml, Peprotech, USA), alone or in combination for the indicated time points and subsequently used for the indicated experiments. Additionally, the JAK inhibitor tofacitinib (0.25 μM; Millipore Sigma, USA) was used. Various in vitro techniques were used to characterize treatment-induced changes to endothelial monolayers. Briefly, flow cytometry was utilized to determine the surface expression of inter-cellular adhesion molecules ICAM-1, E-selectin, and VCAM-1, as well as the adherens junction protein VE-cadherin. Endothelial barrier integrity was measured using Electric Cell-substrate Impedance Sensing (ECIS) technology, while Western Blotting and immunofluorescence analysis were utilized for pathway characterization and visualization. These methods are described in detail in the supplementary data section.

### Flow cytometry analysis of surface ICAM-1, VCAM-1, E-selectin, and VE-cadherin

HAoECs were treated as indicated and collected from the cell culture plates using the PromoCell split kit per the manufacturers’ recommendation. HAoECs were placed in a 12-well round bottom plate and processed as described previously^73^. The following antibodies were used: anti-ICAM-1-BV421 (clone HA58; BioLegend, USA), anti-VCAM-1-PE/Cy7 (clone STA; BioLegend, USA), anti-E-selectin-PE (clone HCD62E; BioLegend, USA), and anti-VE-cadherin-FITC (clone 55-7H1; Fisher Scientific, USA). Assay internal controls and gating were performed using appropriate isotype controls and unstained samples.

### Evaluation of endothelial barrier integrity and immunofluorescence analysis

Endothelial barrier integrity/function was measured on confluent monolayers using Electric Cell-substrate Impedance Sensing (ECIS) technology, and HAoECs were visualized using immunofluorescence as described previously^73^. Initial imaging to visualize gap presence and VE-cadherin expression was done by taking images of the VE-cadherin staining at the same exposure times throughout one set of experiments using an EchoRevolve microscope. Intercellular gaps and VE-cadherin distribution were quantified using the ImageJ analysis program. High-resolution imaging was done using the 780 Zeiss Confocal Microscope located in the NHLBI Light Microscopy Core. Immunofluorescence staining of phosphorylated proteins was performed per the manufacturers’ recommendation. Images were taken with the Echo Revolve microscope.

The following antibodies were used: anti-VE-cadherin (clone F-8; Santa Cruz), p-Stat1 (Tyr701, clone 58D6; Cell Signaling), and p-Stat3 (Tyr 705, clone D3A7; Cell Signaling), all at 1:100 dilution. F-actin was labeled using AF-488-Phalloidin (Thermo Fisher Scientific), while nuclei were labeled using DAPI.

### Western blot

After incubation, HAoECs were lysed using RIPA buffer with protease/phosphatase inhibitors, sonicated, and centrifuged at 1000 × *g* for 5 min at 4 °C. Protein concentration was determined using a standard BCA assay. Equal amounts of protein per sample were separated using SDS-PAGE and transferred to a nitrocellulose membrane using Chameleon Duo Pre-stained Protein Ladder (Licor, USA), blocked using Intercept TBS Blocking Buffer (Licor, USA) for 1 h at room temperature, followed by incubation with primary antibody overnight at 4 °C. Membranes were washed 3 times using 0.1% TBS-Tween-20, and incubated with appropriate secondary antibodies (Licor, USA) for 1 h at room temperature. Subsequently, blots were thoroughly washed using 0.1% TBS-Tween-20 and imaged using a Li-Cor Odyssey imaging system. Western blot analysis was performed using ImageJ of the fluorescence images and Excel software. To determine the activation status of proteins, the fluorescence intensity value of the phosphorylated band was normalized to the corresponding band of the non-phosphorylated protein. Results are shown as fold change of the ratio to the untreated control and displayed as black and white images. GAPDH was used as an assay internal loading control.

All antibodies were diluted in 1:1 Intercept TBS Blocking Buffer: 0.1% TBS-Tween-20: GAPDH (1:10,000; ProteinTech, USA); NFκB (clone D14E12), p-NFκB p65 (S536, clone 93H1), Stat1 (clone 42H3), p-Stat1 (Tyr701, clone 58D6), Stat3 (clone 124H6), p-Stat3 (Tyr 705, clone D3A7) all 1:1,000 dilution and obtained from Cell Signaling Technologies (USA).

### Statistical analysis

In the past, we and others have shown that cytokines may act synergistically in endothelial cells^74–76^, and a potential role for synergistic effects of catecholamines and cytokines has been speculated^77^. Therefore, we explored the product of each cytokine with each catecholamine as a measure of synergism in a correlation analysis. The two cytokines IL-1β and TNFα, the catecholamines epinephrine and norepinephrine, as well as their cytokine/catecholamine products (as a measure of synergism), were subjected to Pearson Correlation analysis. *P* values of < 0.05 are reported as statistically significant in parentheses following the Pearson correlation coefficient (r) value. Subsequently, the p-value was adjusted for multiple comparisons testing using the Holm-Šídák method using STATA release 12 (StataCorp., College Station, TX, USA).

To determine associations between subclinical CVD in our study participants and their serum soluble VE-cadherin levels, multivariable regression analysis was performed using STATA release 12 (StataCorp., College Station, TX, USA). The overall code can be found in Supplementary Fig. 3. Standardized betas with and without adjustment for BMI and/or 10-year ASCVD risk are shown, followed by the p-value in parentheses.

All laboratory-related data were analyzed using Microsoft Excel and PRISM 7.0 (GraphPad) software. The Shapiro-Wilk Test was used to test normality assumptions to determine subsequent statistical models. The null hypothesis was rejected at *P*-value < 0.05. Depending on the dataset’s nature, we performed analyses as follows: For normally distributed data, we used the t-test, paired t-test, one-way ANOVA with Dunnett correction (unpaired), or mixed-effects analysis with Geisser-Greenhouse and Dunn’s corrections (paired). For nonparametric data, we applied the Mann-Whitney test, Kruskal-Wallis test (unpaired), or Friedman test (paired), each with Dunnett or Dunn’s corrections for multiple comparisons. All results are reported as mean ± standard deviation (SD), with exact sample sizes (n) detailed in the figure legends.

## Supplementary information

Supplementary Information

Supplementary Figure 4

## Data Availability

The data pertaining to this study are available from the corresponding author upon reasonable request and within the IRB guidelines for the study protocol.

## Code availability

The overall script of the code utilized in this study can be found in Supplementary Figure 3. The corresponding author can provide any further details.
