# Supplementary material for: Loneliness associates with endothelial dysfunction in a community-based cohort: a pilot study and translational approach
Source: NPJ Cardiovasc Health. 2025 Jun 26;2:28. doi: 10.1038/s44325-025-00059-5 (PMC12885954; doi:10.1038/s44325-025-00059-5)
Supplement: Supplementary file 1 — Supplementary Information [file 44325_2025_59_MOESM1_ESM.pdf]

Supplementary Data

Supplementary Table 1. Baseline characteristics of Washington, DC Cardiovascular Health and Needs Assessment (DC-CHNA) cohort, 2015-2019 (n=42). Continuous variables are reported as Mean ± SD, while categorical variables are shown as N (%).

	DC-CHNA cohort (n=42)
**Demographics and medical history**	
Age, years	60.61 ± 10.56
Female, N (%)	38 (90.5)
Race, African American, N (%)	42 (100)
Body mass index, kg/m2	32.93 ± 8.05
Smoking history, N (%)	15 (37.5)
Hypertension, N (%)	28 (70)
Diabetes mellitus, N (%)	9 (22.5)
ASCVD 10yr risk score	11.19 ± 9.32
**^{18}FDG-PET/CT Measures**	
Aortic Vascular Inflammation	1.627 ± 0.215
Amygdala activity	1.111 ± 0.087
**Biomarkers & Cytokines**	
TNFα, pg/mL	1.53 ± 0.46
IL-1β, pg/mL	0.17 ± 0.09
Epinephrine, pg/ml	30.56 ± 15.26
Norepinephrine, pg/ml	441.11 ± 262.00
Serum VE-cadherin	2325.44 ± 646.66

Acronyms: ASCVD-Atherosclerotic Cardiovascular Disease 10-year risk score; DC-CHNA – Washington, DC Cardiovascular Health and Needs Assessment

Supplementary Table 2: Pearson correlation analysis of self-reported measures of psychosocial stressors to IL-1β and TNFα, norepinephrine (NE), and each of their products. Results are shown as the Pearson correlation coefficient (r) value followed by the p-value in parenthesis with the multiple comparison-adjusted p-values in brackets. Significance is indicated in bold font and an asterisk (*) when a p-value <0.05 is reached.

				**Synergism**	
	**Norepi (NE)**	**IL-1β**	**TNFα**	**NE and IL-1β**	**NE and TNFα**
Social Isolation#	-0.19 (0.23) [0.54]	0.21 (0.17) [0.43]	0.10 (0.54) [0.90]	0.14 (0.37) [0.75]	0.03 (0.87) [0.99]
Loneliness#	-0.01 (0.97) [1.00]	0.04 (0.83) [0.99]	0.25 (0.12) [0.31]	0.05 (0.77) [0.99]	0.23 (0.14) [0.36]

#Loneliness, Social Isolation – increasing values indicate an increasing measure of chronic psychosocial stress

Supplementary Figures

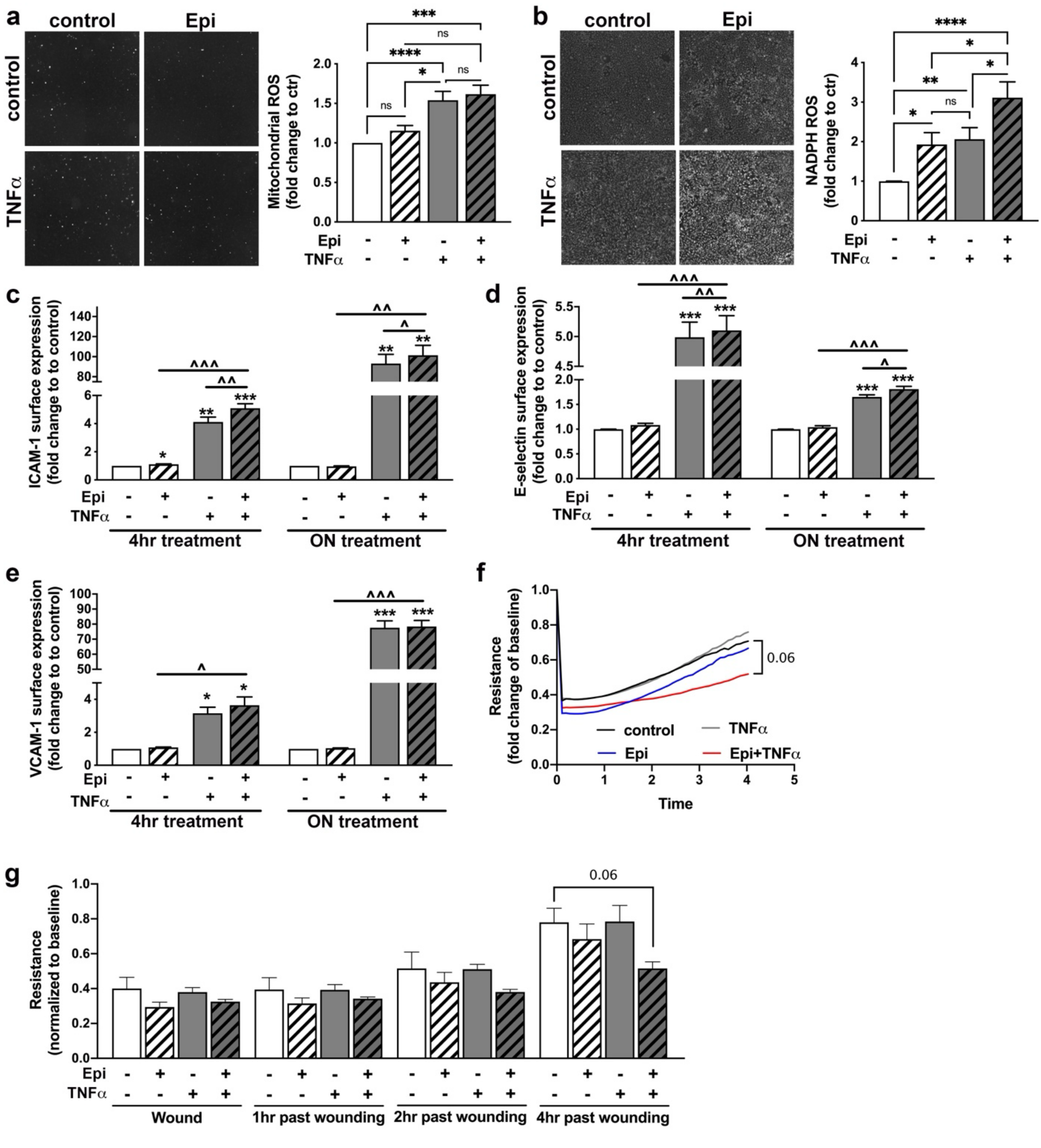

Supplementary Figure 1. Epinephrine and TNFα combinatory treatment induces pro-atherosclerotic endothelial phenotype. HAoEC were treated as indicated. **(a)** Mitochondrial ROS were labeled using Mitosox after overnight treatment. Representative images are shown on

the left. (n=6, Friedman test with Dunn's correction) **(b)** NADP-dependent ROS were labeled using the DCFDA after overnight treatment. Representative images are shown on the left of the panel. (n=6, Friedman test with Dunn's correction) **(c-f)** Intercellular adhesion molecules ICAM-1, VCAM-1 and E-selectin were detected using flow cytometry at the indicated timepoints with stated treatments. (n=5-6; RM one-way ANOVA analysis with Tukey correction) (**f/g**) ECIS was used to measure recovery of endothelial barrier resistance after wounding subsequently to overnight treatment as indicated. (**f**) Summary curve of individual experiments. (**g**) Presentation of resistance changes at indicated timepoints past wounding. (n=4, Friedman test with Dunn's correction) (* indicates significance to corresponding control, ^ indicates significance between indicated groups; Abbreviations: ctr=control, ECIS=Electric Cell-Substrate Impedance Sensing, Epi=epinephrine, HAoEC=human aortic endothelial cells, hr=hours, ON=overnight, TNFα=tumor necrosis factor-alpha)

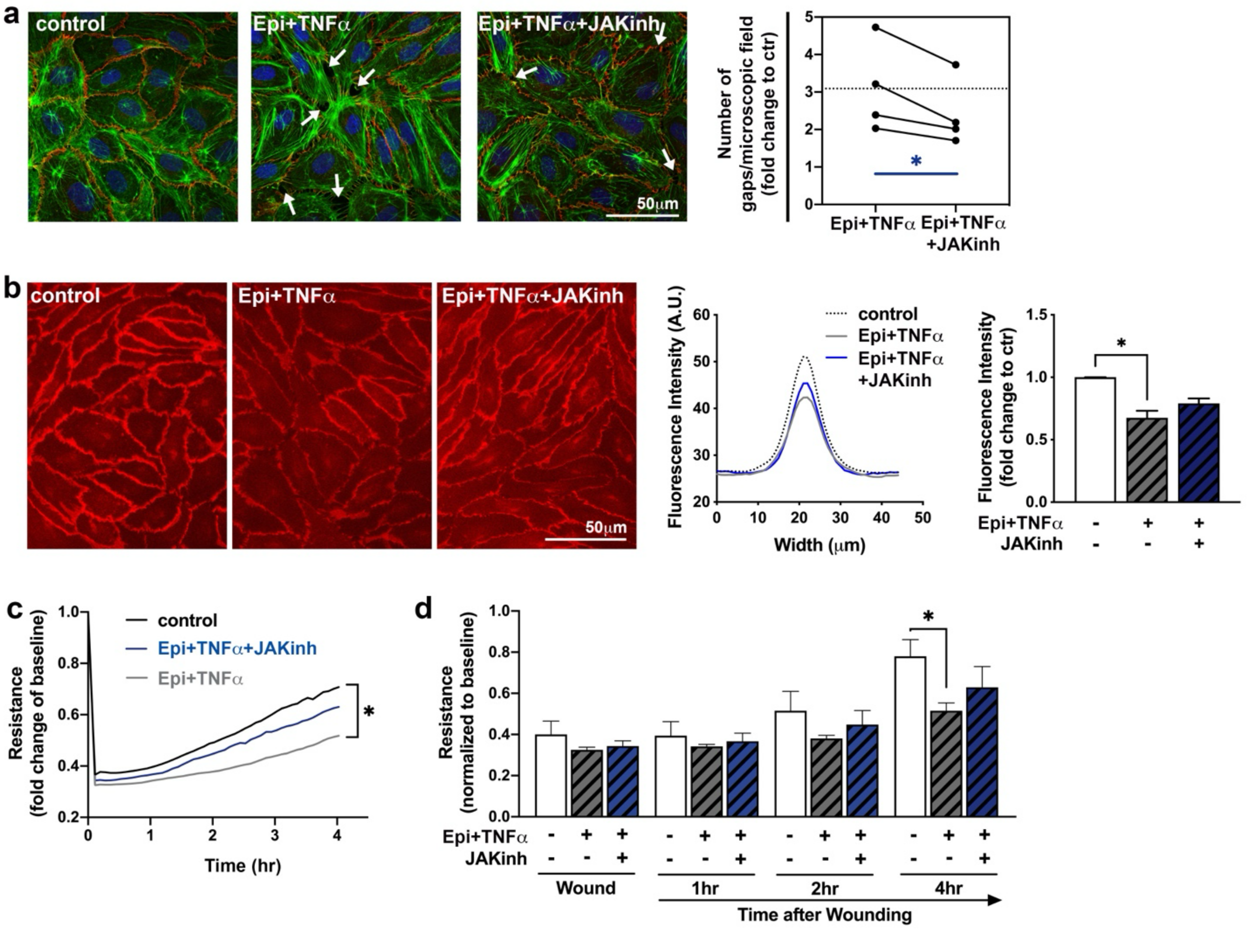

Supplementary Figure 2. Impact of JAK/Stat pathway inhibition on Epi+TNFα-induced endothelial dysfunction – after 6 hours of treatment. HAoECs were treated with control, Epi+TNFα, or Epi+TNFα in the presence of tofacitinib, a JAK/Stat pathway inhibitor for 6hr. (**a**) Immunofluorescence staining of VE-cadherin (red) and labeling of F-actin (green) as well as the nuclei (blue) with subsequent quantification of intercellular gap formation (n=4; paired t-test between Epi+TNFα and Epi+TNFα+JAK inhibitor). White arrows indicated gaps between endothelial cells. **(b)** Images of VE-cadherin immunofluorescence after 6hr treatment were taken at equal exposure time per set, and the fluorescence intensity over the junctional space was measured as displayed in the histogram. Subsequent quantification of n=5 is displayed in the graph (Kruskal-Wallis test with Dunn's correction). **(c/d)** ECIS was used to determine recovery of

endothelial barrier resistance after wounding, subsequently to overnight treatment as indicated. (**c**) Summary curve of individual experiments. (**d**) Presentation of resistance changes at indicated timepoints past wounding. (n=4, Friedman test with Dunn's correction) (* indicates significance $p<0.05$, ** indicates significance $p<0.01$; Abbreviations: ECIS=Electric Cell-Substrate Impedance Sensing, Epi=epinephrine, HAoEC=human aortic endothelial cells, hr=hours, ON=overnight, TNFα=tumor necrosis factor-alpha)

a Pearson Correlation

DoFile_Table3 Pearson2

```
log using "Table3 Pearson2"

set more off
foreach var in icam1mfi vcam1mfi eselectinmfi vecadherinmfi{
    pwcorr `var' loneliness, sig star(.05)
    pwcorr `var' loneliness, sig star(.05) sidak
}
log close
```

b Multivariable Linear Regression Analysis

```
log using "sVE to VI"
set more off
foreach var in avtbrall {
     regress `var' vecadherinngml, b
     regress `var' vecadherinngml bmikgm2 , b
     regress `var' vecadherinngml ascvd10yr, b
     regress `var' vecadherinngml bmikgm2 ascvd10yr, b
}
log close
```

Supplementary Figure 3. STATA example codes utilized in this study. (a) Displays the code for Pearson Correlation, while (b) displays the code for multivariable regression analysis.
