# Supplementary material for: Loneliness associates with endothelial dysfunction in a community-based cohort: a pilot study and translational approach
Source: NPJ Cardiovasc Health. 2025 Jun 26;2:28. doi: 10.1038/s44325-025-00059-5 (PMC12885954; doi:10.1038/s44325-025-00059-5)

Supplementary Figure 4A. Uncropped Western Blot images to Figure 2b, c, and e. Western Blots were cut to allow probing of several proteins with different molecular weights. Phospho-proteins were analyzed in the first step, while unphosphorylated proteins were analyzed after stripping and re-staining process. Data were analyzed as the ratio of Phospho-protein over unphosporylated protein and displayed as the mean fold change to control/vehicle treatment.

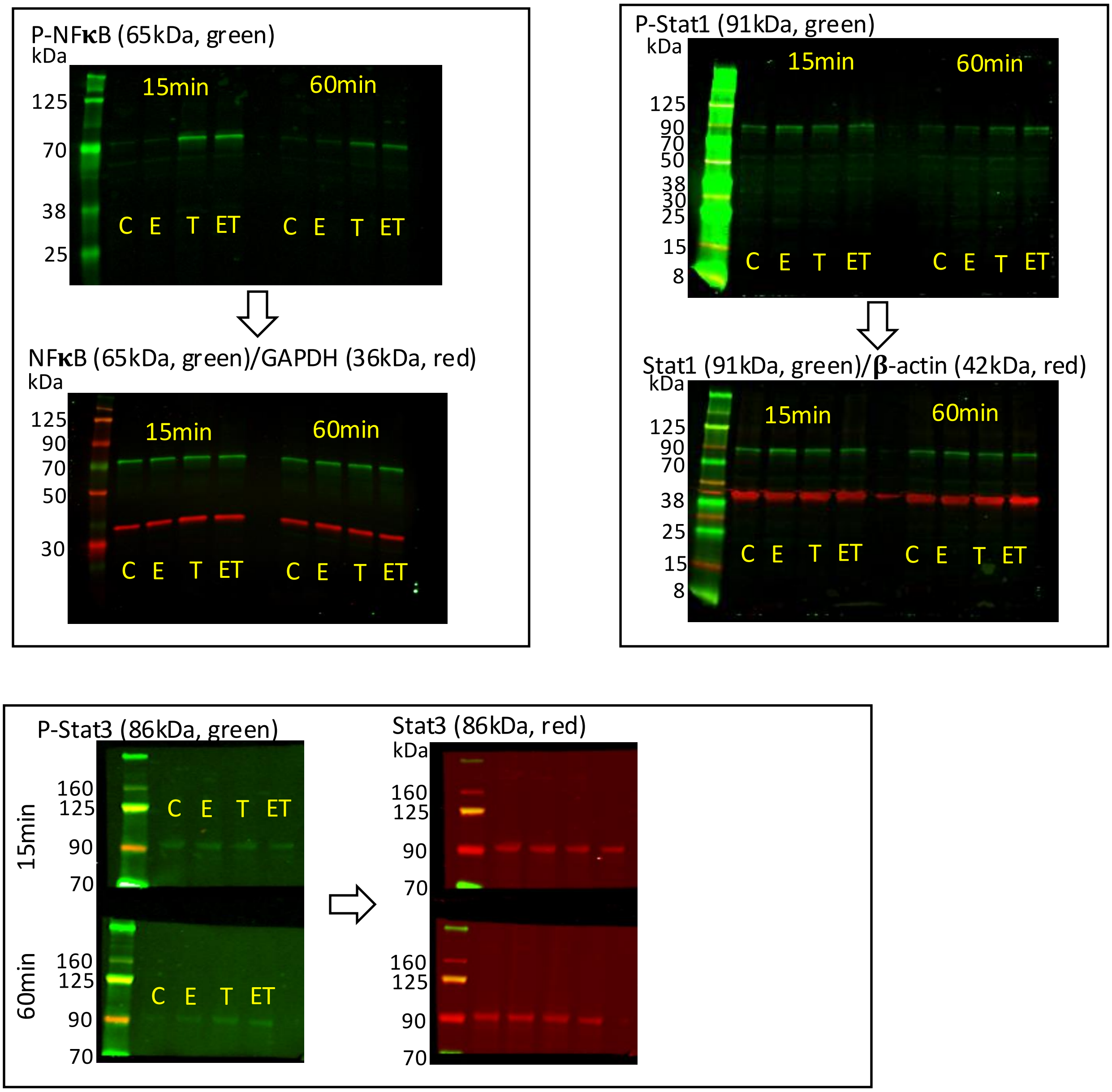

Supplementary Figure 4B. Uncropped Western Blot images to Figure 2g/h. Western Blots were cut to allow probing of several proteins with different molecular weights. Phospho-proteins were analyzed in the first step, while unphosphorylated proteins were analyzed after stripping process. Data were analyzed as the ratio of Phospho-protein over unphosporylated protein and displayed as the mean fold change to control/vehicle treatment.

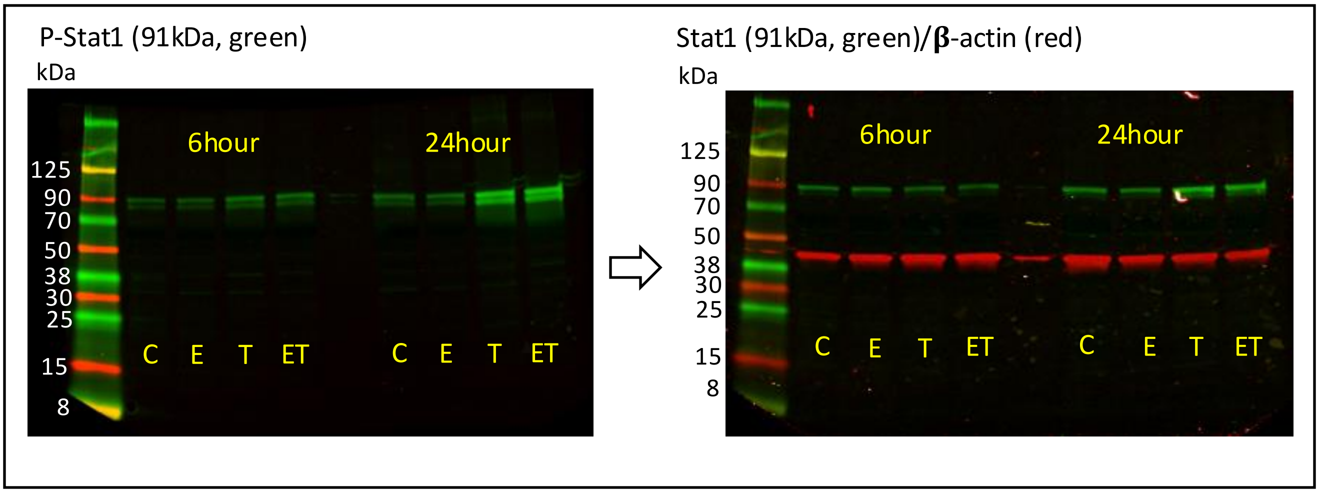

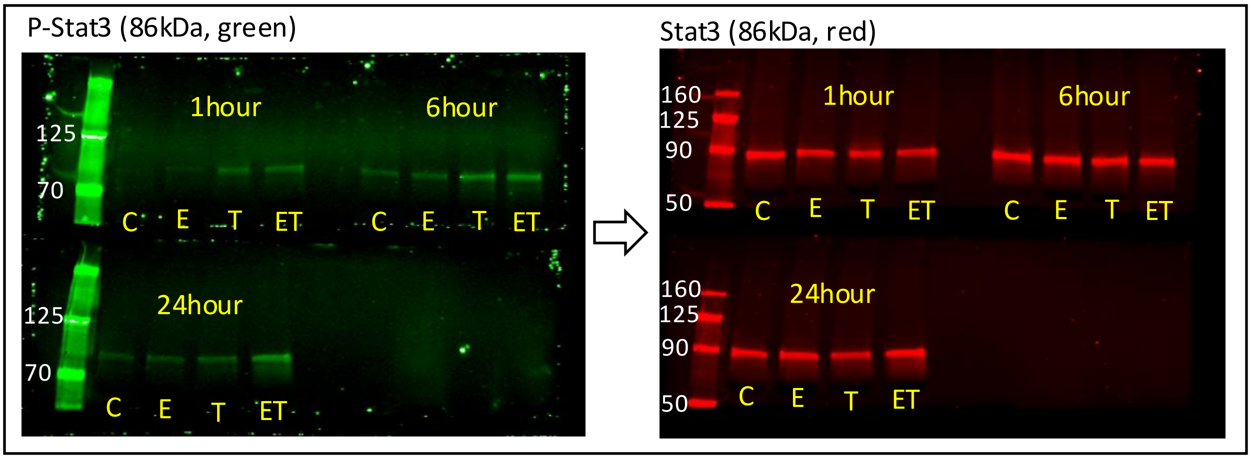

Supplementary Figure 4C. Uncropped Western Blot images to Figure 3b. Western Blots were cut to allow probing of several proteins with different molecular weights. Phospho-proteins were analyzed in the first step, while unphosphorylated proteins were analyzed after stripping process. (Abbreviations: i-inhibitor for indicated pathway) →only the JAK inhibitor was included in the manuscript. Data were analyzed as the ratio of Phospho-protein over unphosporylated protein and displayed as the mean fold change to control/vehicle treatment.

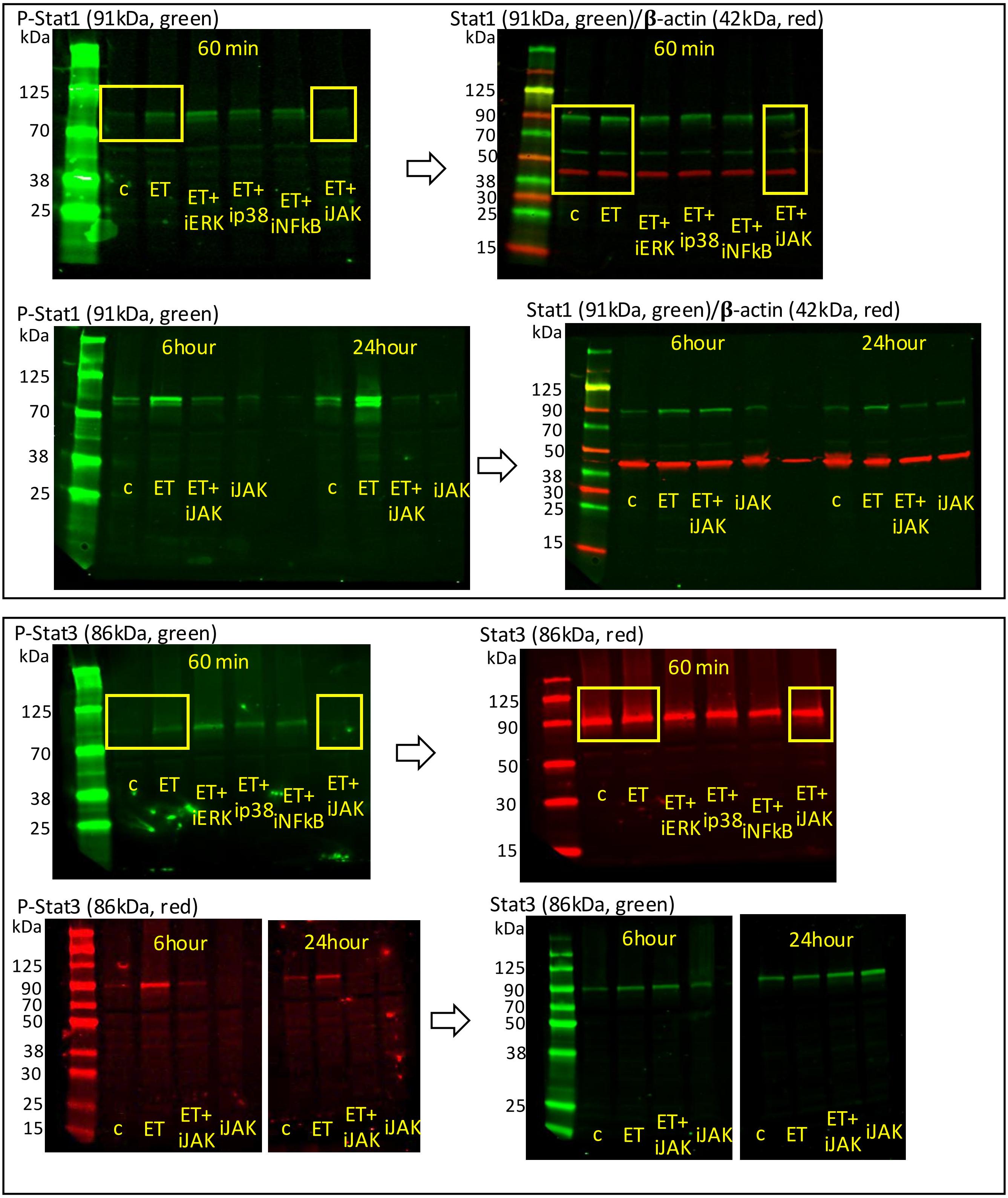

Supplement: Supplementary file 2 — Supplementary Figure 4 [file 44325_2025_59_MOESM2_ESM.pdf]
